# Analysis of the Prevention of Mother-to-Child Transmission (PMTCT) Service utilization in Ethiopia: 2006-2010

**DOI:** 10.1186/1742-4755-8-6

**Published:** 2011-04-16

**Authors:** Tilahun Nigatu, Yoseph Woldegebriel

**Affiliations:** 1 Health, AIDS, Population and Nutrition Office; USAID/Ethiopia; P.O. Box 1014; Addis Ababa, Ethiopia

## Abstract

**Introduction:**

Although progressive improvements have been made in the coverage and quality of prevention of HIV/AIDS mother-to-child transmission (PMTCT) services in Ethiopia, the national coverage remained persistently low. Analysis of the cascaded PMTCT services can reveal the advancements made and the biggest hurdles faced during implementation.

**Objective:**

To examine the progresses and unaddressed needs in access and utilization of PMTCT services in Ethiopia from 2006 to 2010 thereby developing best-fit regression models to predict the values of key PMTCT indicators at critical future points.

**Methods:**

Five-year national level PMTCT data were analyzed in a cascaded manner. Five levels of analysis were used for ten major PMTCT indicators. These included description of progress made, assessment of unaddressed needs, developing best-fit models, prediction for future points and estimation using constant prevalence. Findings were presented using numerical and graphic summaries.

**Results:**

Based on the current trend, Ethiopia could achieve universal ANC coverage by 2015. The prevalence of HIV at PMTCT sites has shown a four-fold decrease during the five-year period. This study has found that only 53% of known HIV-positive mothers and 48% of known HIV-exposed infants have received ARV prophylaxis. Based on assumption of constant HIV prevalence, the estimated ARV coverage was found to be 11.6% for HIV positive mothers and 8.4% for their babies.

**Conclusion:**

There has been a remarkable improvement in the potential coverage of PMTCT services due to rapid increase in the number of PMTCT service outlets. However, the actual coverage remained low. Integration of PMTCT services with grassroots level health systems could unravel the problem.

## Introduction

According to the latest data, significant progress has been made in delivering prevention of mother-to-child transmission of HIV services in low and middle income countries. However, much work remains to be done. An estimated 430,000 children were newly infected with HIV in 2008, the vast majority of them through mother-to-child transmission [1].

According to the 2010 report, *Towards universal access: scaling up priority HIV/AIDS interventions in the health sector *by the World Health Organization (WHO), significant progress in the area of PMTCT has been made during the past several years. In 2009, 53% [40-79%] of the estimated HIV-infected pregnant women worldwide received at least some antiretroviral (ARV) drugs to prevent HIV transmission to their child [2].

With a national adult HIV prevalence of 2.1%, Ethiopia is one of the countries most severely hit by the epidemic. Besides the dominant heterosexual transmission, vertical virus transmission from mother-to-child accounts for more than 90% of pediatric AIDS. It is estimated that over 90% of childhood HIV infections result from the transmission of the virus from mothers to their children during and soon after birth [3,4].

Although the Government of Ethiopia has given high priority to PMTCT, and progressive improvements have been made in the coverage and quality of PMTCT services, the national coverage remained persistently low [5,6] and [7]. In 2010, five years after large scale implementation of PMTCT programs, only 6,990 HIV+ pregnant mothers received ARV prophylaxis. This was just 18.7% of the annual planned target [8]. Analysis of the cascaded PMTCT services can reveal the advancements made and the biggest hurdles faced during the implementation [9]. Furthermore, such analysis can highlight future directions of program outcomes and uncover factors of important consideration for enhanced future achievements.

Studies has shown that various socio-cultural, health systems, basic infrastructure, and other challenges contribute to poor maternal health services in general and low PMTCT service coverage in particular in most developing countries including Ethiopia. However, the envisaged study was limited in analyzing data compiled from service delivery points to reveal specific progresses and trends in the PMTCT service cascade.

Moreover, it is believed that if data is properly collected, analyzed, and interpreted, it can be used for decision making at different levels of the health system. At regional and national levels, data collected and compiled from service delivery points can inform policy changes. Since this study is primarily based on analysis of secondary data, it can help health professionals, program managers, and policy makers view the trends and prospects of key PMTCT indicators.

Therefore, the aim of this study was to examine the progresses and unaddressed needs in access and utilization of PMTCT services in Ethiopia from 2006 to 2010 thereby developing best-fit regression models to predict the values of key PMTCT indicators at critical future points.

## Methods

### Study area and period

This study presents relevant PMTCT evidence from Ethiopia in an aggregated manner. Based on the single point estimate of HIV/AIDS prevalence of HAPCO, Ethiopia has an estimated HIV prevalence of 2.4% with more than 1 million people living with HIV in 2010. The HIV epidemic in the country is generalized, but with concentrations at specific most at risk populations. This analysis was performed in Jan 2011.

### Study design

A time-series and cascaded analysis of PMTCT data was conducted on national level PMTCT data for the period of 2006 to 2010.

### Study populations

The reference population for this study was the population of all pregnant mothers in Ethiopia between 2006 and 2010. The study population thus was all pregnant mothers with antenatal care visits during the five-year study period. There could be multiple counting of study subjects; a mother may have more than one pregnancy during the five-year study period.

### Sample and sampling

Sampling in this study was generally purposive. The five-year study period (2006 to 2010) was used based on the availability of well-organized and published PMTCT data. The selection of the indicators/variables was based on their direct relevance to the subject of analysis. A total of ten key variables were selected for the analysis (Table 1).

**Table 1:** The ten key indicators of PMTCT service cascade included in the study, Jan. 2010

#	Indicator	Description
**1**	Estimated number of expected pregnancies	Annual number of expected pregnancies in the country calculated as a proportion from the total population

**2**	Antenatal care coverage	The proportion of pregnant mothers with at least one ANC visit during their pregnancy in the fiscal year

**3**	Number of PMTCT sites	The total number of health service delivery outlets reported to be providing PMTCT services during the reporting period

**4**	Proportion of ANC at PMTCT sites	The proportion of ANC attendees who received ANC services at sites providing PMTCT services

**5**	Proportion of mothers counseled for HIV	The proportion of ANC attendees at PMTCT sites who were counseled for HIV

**6**	Proportion of mothers tested for HIV	The proportion of HIV counseled mothers who have consented for HIV testing and known their status at PMTCT sites

**7**	Proportion of HIV+ mothers	The proportion of HIV tested mothers whose results were reported to be reactive from PMTCT sites

**8**	Proportion of HIV+ mothers received ARV	The proportion of HIV+ mothers who received ARV (SDNVP, combined ARV) from PMTCT sites

**9**	Proportion of HEI received ARV	The proportion of HIV exposed infants (HEI) who received ARV (calculated as a proportion of HIV+ pregnant women)

**10**	Estimated number of HIV+ pregnant women	Estimated based on HIV prevalence at PMTCT sites and on the assumption that the prevalence of HIV is the same for all pregnant mothers

### Data collection

This study used only secondary data collected from published documents. Data on national level PMTCT indicators was collected mainly from three major series of publications from the Federal Ministry of Health (FMOH) and Federal HIV/AIDS Prevention and Control Office (HAPCO) of Ethiopia, all published on a regular basis:

○ Health and Health related indicators;

○ Annual HIV/AIDS Monitoring and Evaluation Report; and

○ AIDS in Ethiopia Surveillance Reports.

### Data analysis and interpretation

After data collection from these public documents was completed, they were organized into a spreadsheet and the following levels of analysis in MS Excel 2010 and STATA 10.0 were preformed:

○ **Description of progress**. Progress in access and utilization of PMTCT services across the PMTCT cascade were described using numbers and percentages with graphic and tabular presentation. Reported values of PMTCT indicators were used directly at this stage of analysis.

○ **Assessment of unaddressed needs**. Unaddressed needs were identified and measured by calculating the difference between the number of eligible service recipients expected at each level of the cascade and the actual number of recipients at the same level of the cascade. These were described in actual numbers and percentages and in some cases stratified for service and time units.

○ **Development of best-fit models**. Best-fit models, which can adequately explain the nature of the data across time, were identified using regression equations. The level of fitness of the model was determined using R-squared. Linear and exponential equations were used in this study.

○ **Prediction for future status**. Expected values of some indicators at critical future points in time were predicted using the regression equations identified assuming that the same trend will continue. Future points were used to predict the indicator values at those points and in other cases expected indicator values were used to calculate the future time point at which the specified indicator value can be attained.

○ **Estimation of coverage**. Constant HIV prevalence assumption was used to estimate the annual number of HIV+ pregnant women, which was further used to estimate the proportion of HIV+ pregnant women identified and the ARV prophylaxis coverage for HIV+ pregnant women and HIV exposed infants.

## Results

### Antenatal care (ANC) coverage

**Figure 1:**
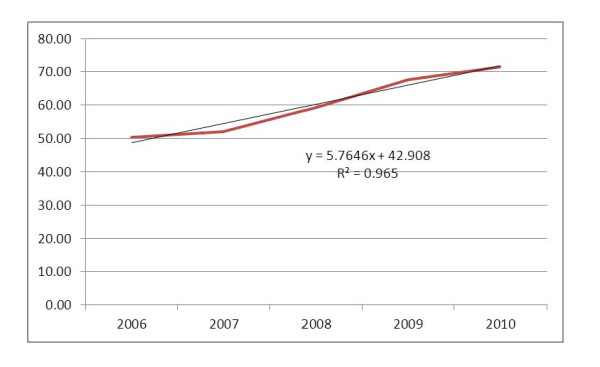
**Progress in antenatal care service coverage (%) in Ethiopia: 2006-2010**.

The antenatal care service coverage for at least one visit during pregnancy has increased from 50% in 2006 to 71% in 2010. This service coverage has been increasing by an average of 5.16% per year. The highest annual increment was in 2008 which was 8.3%. In contrast, the annual number of pregnant mothers who have not received any ANC service has declined from 1.4 million in 2006 to 845,298 in 2010. The rate of decline is about 150 thousand per year. The overall progress of the ANC coverage over the five years period follows a linear trend and can be described by the trend-line equation displayed in Figure 1. If the same trend is assumed to continue, prediction using the trend-line equation shows that universal coverage for ANC (100% coverage) can be achieved in 2015 (Figure 1).

### Potential PMTCT service coverage

**Figure 2:**
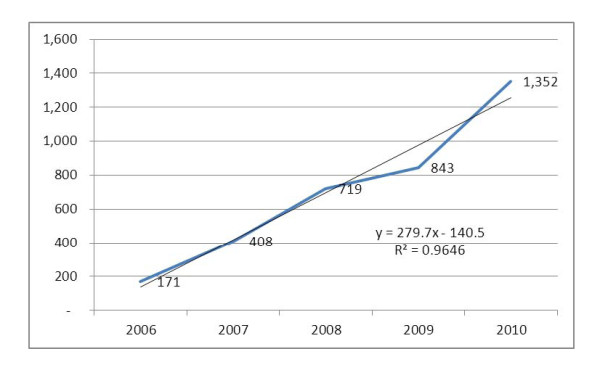
**Expansion in the number of PMTCT sites in Ethiopia: 2006-2010**.

During the five year period, the number of health facilities providing PMTCT services has shown an 8-fold increment from 171 (21.3%) in 2006 to 1,352 (61.9%) in 2010 with an average rate of 236 new PMTCT sites a year. The Health Sector Development Plan IV for Ethiopia stipulates that 100% of health centers and hospitals will be providing PMTCT services by 2015. Given that the current trend in the expansion of PMTCT sites is constant, there will be 2,657 PMTCT sites by 2015 (Figure 2).

**Figure 3:**
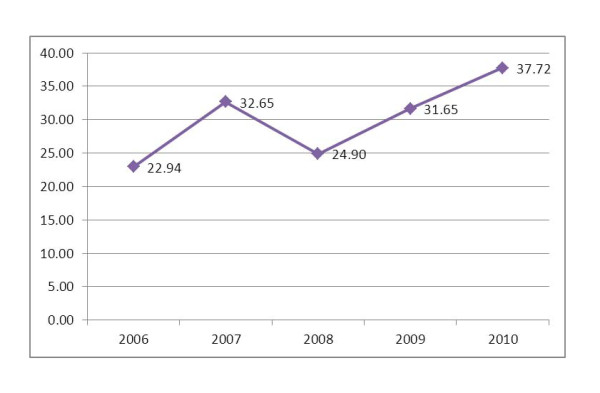
**Progress in the proportion/percent of ANC at PMTCT sites: 2006-2010**.

The proportion of ANC clients receiving services at PMTCT sites, based on the percentage of ANC at PMTCT sites, has increased from 22% in 2006 to 38% in 2010. However, the pattern of increment was not linear. In reviewing the overall ANC coverage during the study period, there was a decline from 32.6% in 2007 to 24.9% in 2008 and this was followed by a linear increment as of 2009 (Figure 3).

Despite the expansion of PMTCT sites and an increase in the number of mothers receiving ANC at PMTCT sites, an annual average of 1.3 million pregnant mothers were attended for ANC at sites which did not have PMTCT service during the five-year period. The highest number of ANC visits at non-PMTCT sites was recorded to be 77% in 2006 and 75% in 2008. This shows that there was a disproportional increase of ANC attending mothers at PMTCT and non-PMTCT sites during the five-year period.

### HIV Counseling at PMTCT sites

**Figure 4:**
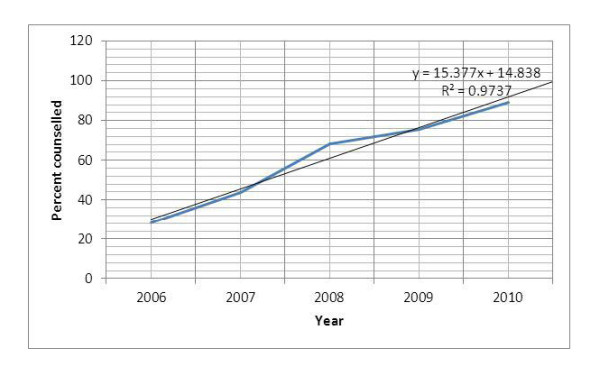
**Proportion of ANC clients at PMTCT sites provided with HIV counseling services**.

A three-fold increase was observed in the proportion of ANC clients provided with HIV counseling services at PMTCT sites. As shown below, this has followed a linear trend. Based on the trend-line equation, 100% coverage in counseling can be achieved in 2011 (Figure 4). Correspondingly, the annual number of pregnant mothers who received ANC at PMTCT sites, but have not been counseled for HIV has decreased from about 230,000 in 2006 to 85,000 in 2010. Based on the 2010 reported figure, every PMTCT site missed an average of 63 pregnant mothers present for ANC; every workday an average of 326 pregnant mothers present for ANC missed HIV counseling services.

### HIV testing at PMTCT sites

Significant progress has been made in the proportion of pregnant mothers who accept HIV testing after receiving counseling services for HIV. At an average yearly increment rate of 7%, the proportion of HIV counseled mothers who accepted HIV testing has increased from 57% to 92%. Numerically, the number of mothers who accepted HIV testing has increased from 52,428 in 2006 to 653,065 in 2010. On the other hand, a total of about 300,000 mothers who received HIV counseling services did not test for HIV during the five-year period. This was about 17% of the total mothers counseled for HIV. In other words, every year about 60,000 mothers counseled for HIV decide not to test for HIV. This is equivalent 48 mothers per facility per year and 232 mothers per working day.

**Figure 5:**
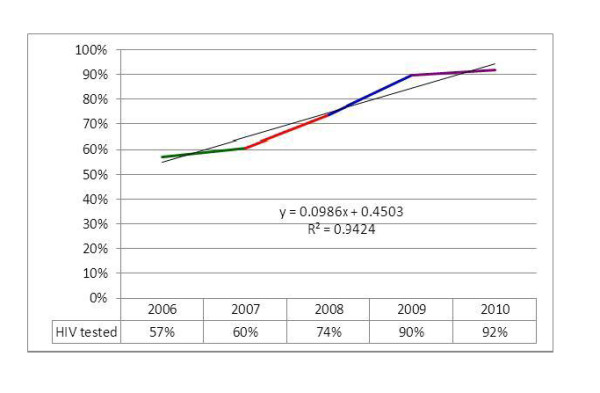
**Proportion of counseled PMTCT clients provided with HIV testing services**.

This is more than a 12-fold increment in the number of ANC mothers counseled for HIV within the five year period. Keeping all other things constant and using the trend-line equation for prediction, 100% testing of all mothers counseled for HIV could be achieved within one year forward from 2010 i.e. 2011 (Figure 5).

### HIV Prevalence

**Figure 6:**
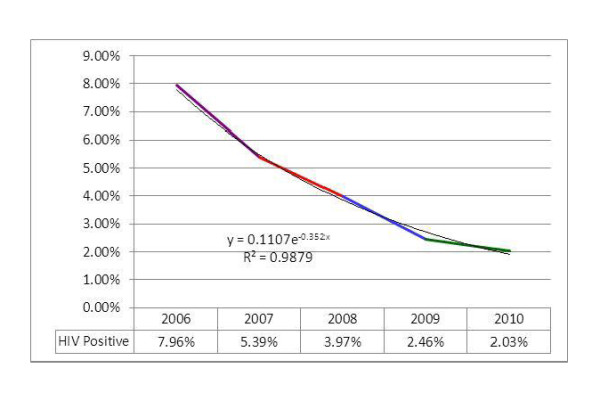
**Proportion of HIV positive pregnant women among those who received HIV test at PMTCT sites**.

As it can be seen from the figure 6, the prevalence of HIV among those pregnant mothers who underwent HIV testing has decreased from 8% to 2% within the reporting period. The pattern of reduction in HIV prevalence followed and exponential pattern. Although the prevalence has shown a significant reduction, the number of HIV positive pregnant mothers identified per year has increased from 4,172 in 2006 to 13,257 in 2010. During the entire five-year period, a total of 42,195 HIV positive mothers were identified out of the 1,462,565 mothers tested for HIV making the overall prevalence of HIV 2.9%. Using the current trend-line equation for prediction, the prevalence of HIV among pregnant women at ANC sites in 2015 will be 0.33% (Figure 6).

### ARV Prophylaxis

**Figure 7:**
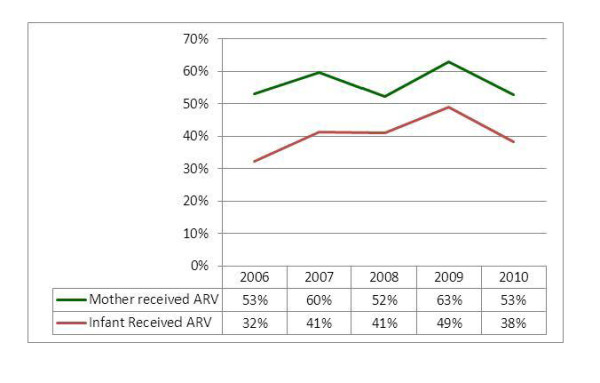
**Proportion of HIV+ pregnant women and HIV exposed infant who received ARV**.

For the study period, the proportion of HIV positive pregnant women who received ARV prophylaxis is higher than the corresponding annual figure for the proportion of infants born to HIV mothers who received ARV prophylaxis by an average of 16.4%. However, both indicators of ARV use for prophylaxis had a swinging pattern with higher values in 2007 and 2009 and relatively lower values in 2006, 2008 and 2010. Out of the total mothers tested positive for HIV (42,195) during the five-year period, 24,109 (56%) have received ARV prophylaxis. Throughout the study period, 18,806 (44%) of the HIV+ pregnant mothers and 25,260 (59%) of the HIV exposed infants did not received ARV prophylaxis (Figure 7).

### From ANC attendance to HIV test in PMTCT cascade

**Figure 8:**
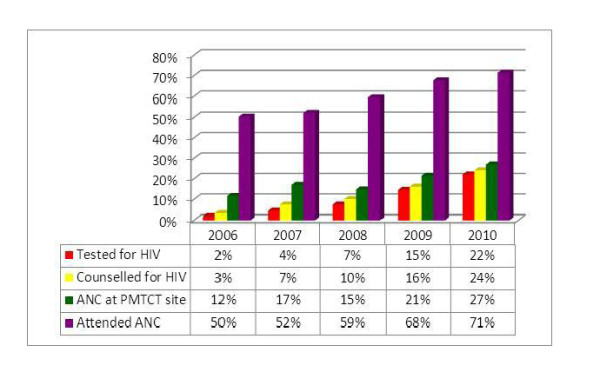
**From ANC attendance to HIV test in the PMTCT cascade of Ethiopia: 2006-2010**.

All the service utilization steps from ANC attendance to the acceptance of HIV testing followed an increasing pattern during the five-year period. As shown in the figure below, the largest hurdle has been in the difference between the proportion of mothers who attended ANC and proportion of mothers who attended ANC at PMTCT sites. Acceptance for HIV testing among those pregnant mothers who were counseled for HIV has shown significant improvement; declining HIV counseling after presenting for ANC at PMTCT sites and refusing HIV testing after counseling have shown progressive reductions during the study period. As indicated in the figure below, the proportion of ANC attendance at PMTCT sites has shown a 15% increase, while all the remaining three rising indicators have shown at least 20% increase during the study period (Figure 8).

### From HIV positive test result to ARV for infants in PMTCT cascade

**Figure 9:**
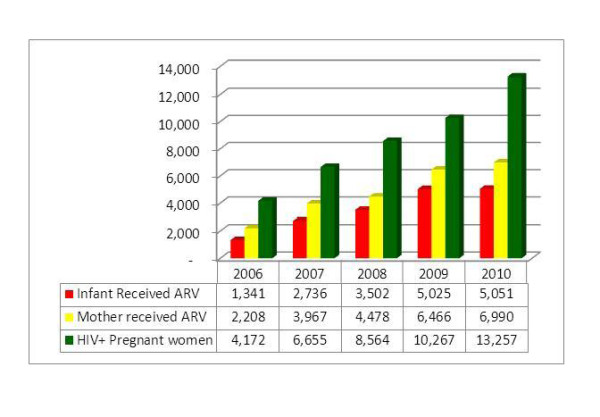
**From HIV positivity to ARV for exposed infant in the PMTCT cascade: 2006-2010**.

As compared to the 2006 figure, the number of HIV positive pregnant mothers identified, the number of HIV positive pregnant mothers who took ARV prophylaxis and the number of HIV exposed infants who received ARV have increased by more than three-fold. However, this seems lower as compared to the eight-fold increment in the number of health facilities providing PMTCT services during the five-year period. It also seems apparently lower as compared to the twelve-fold increase in the number of pregnant women tested for HIV, though it might be difficult to assume constant yield of sero-positivity (Figure 9).

**Figure 10:**
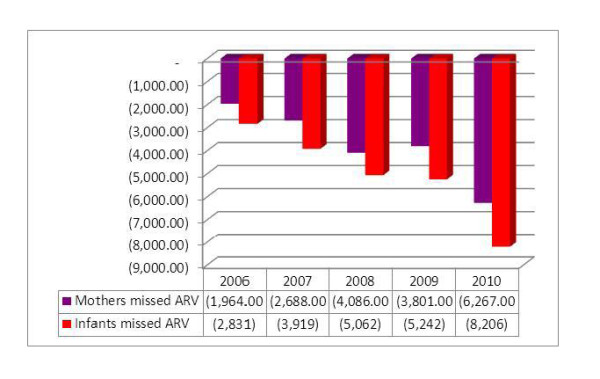
**Number of eligible HIV+ pregnant mothers and HIV exposed infants missed from ARV**.

Despite this fact, the difference between the number of mothers who tested HIV positive and the number of mothers who received ARV had increased from 1,964 in 2006 to 6,267 in 2010, a three-fold increment. Similarly, the difference between the number of mothers who tested HIV+ and the number of HIV exposed infants who received ARV has increased from 2,831 in 2006 to 8,206 in 2010, nearly a three-fold increment. Hence, both arms of the cascade have shown a three-fold increment in units of number of mothers and infants receiving ARV (Figure 10).

### Estimation with constant HIV prevalence

Assuming constant HIV prevalence for all groups of pregnant mothers in the country and using the prevalence from ANC/PMTCT sites as a point estimate, it is possible to estimate the total number of HIV positive pregnant women in the country as proportion of all expected pregnancies. Such estimations are shown in the Table 2.

**Table 2:** Estimated number of HIV+ pregnant women based on constant prevalence

Year	Total expected pregnancies	HIV prevalence	Estimated HIV+ mothers
**2006**	2,794,295	7.96%	222,358

**2007**	2,753,434	5.39%	148,518

**2008**	2,902,330	3.97%	115,151

**2009**	2,877,881	2.46%	70,714

**2010**	2,955,587	2.03%	59,997

**Figure 11:**
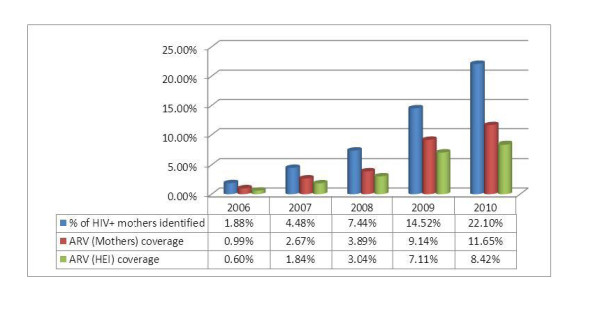
**Cascade of case identification and ARV coverage based on constant prevalence**.

The proportion of annual estimated HIV positive mothers identified at PMTCT sites has increased from 1.88% in 2006 to 22.10% in 2010. Although still at lower levels, the proportion of annual estimated HIV positive pregnant women and their infants who received ARV prophylaxis has undergone significant progresses (Figure 11).

## Discussion

Although there is a significant increasing trend in the number of pregnant mothers attending ANC in Ethiopia, by the end of 2010, 29% were not yet receiving any ANC service. There is no national generalizable study to show the reasons behind this data. However, according to the last Ethiopia Demographic and Health Survey (EDHS 2005), these numbers are attributed to the culture of societies, the poor quality of health services, and the perception and attitude of the health care workers [10,11]. These are serious challenges that need time and well thought-out strategic approaches to make a real and sustainable difference in the health seeking behavior of pregnant women.

This study showed that 62% of mothers are receiving ANC services at non-PMTCT sites. These women have attended ANC services in either health centers that do not offer HIV provider-initiated testing and counseling (PITC) services for pregnant women, or at health posts by Health Extension Workers (HEWs), or rarely in outreach sites [12]. This fact is consistent with the findings of the JSI/L10K project survey which reported that in four major regions of the country, which constitute the biggest populations, about 20% of the women had visited a health post at least once the previous year for antenatal care [13]. The Federal Ministry of Health of Ethiopia allows HEWs in high HIV prevalent regions and "Hot Spot" areas to do HIV counseling and testing (HCT) for pregnant women. However, in most locations this is not the case. This finding has an implication for policy makers and program officers at the regional level; the fact that a large number of pregnant women access HCT through HEWs at the most basic health post level calls for a need to deliver PMTCT services at Health post level. On the contrary, the figure also shows that health facilities are expanding more rapidly than PMTCT sites; this can be considered as missed opportunities for PMTCT services in the country.

The Government of Ethiopia has designed and implemented the opt out HCT strategy in 2005. The acceptance and achievement of this policy can be partly demonstrated by the promising practices observed at PMTCT sites. The national average shows that 89% of pregnant women are counseled for HIV and 92% of those pregnant women who were counseled have accepted the test and were actually tested for HIV/AIDS, while only 8% opt not to be tested.

This research shows that HIV prevalence at PMTCT sites is exponentially declining. There could be many explanations for this decline. One reason may be due to the fact that low risk mothers are getting pregnant, as most of these mothers know their status before pregnancy. It may be also because of low risk mothers consenting for HIV testing or the expansion of PMTCT services in low HIV prevalence zones. More and more pregnant women are getting access to HIV counseling and testing (HCT); this increase can also raise the denominator, making the overall prevalence decline. Of course, an actual decline of HIV prevalence among pregnant women also cannot be ruled out.

The main objective of all PMTCT services remains having needy women and their babies take ARV prophylaxis. According to this review, 47% of known HIV+ pregnant women are not receiving ARVs and 62% of known HIV exposed infants are not receiving ARVs. This result is consistent with the findings of other developing countries [14]. Many reasons can be attributed to this low coverage. The most important reasons include stigma and discrimination, the poor quality of post-test counseling and follow up, high turnover and/or shortage of trained staff, and the elevated level of home delivery; of course, the inconsistent supply of commodities, particularly ARVs, and mal-functioning logistics also have a significant contribution to the problem [11]. The other important factor is the poor documentation and reporting system in the country. For example, the monthly summary reporting form does not capture the HCT and ARVs provided in labor and delivery units and the numbers of HIV positive women who get pregnant while on ART.

A study by Tura in selected health facilities in northwest Ethiopia showed that over 60% of pregnant women visit health facilities for ANC in their first or second trimester, i.e. before the twenty-eighth week of gestation [15]. According to the EDHS 2005, among urban women over 80% of all who attend ANC are less than six months pregnant at the time of their first ANC visit. Even among those women who visit health facilities for ANC services, only about 20% of them get the recommended four ANC visits during their pregnancy [10,13]. If these findings are accurate, then similarly, about 60% of HIV+ women seen at health centers and hospitals come for ANC before their twenty-eighth week of pregnancy.

Based on the above cited literature and WHO estimates, reasonable assumption are that about 60% of HIV+ women enter ANC during their first or second trimester, and that 20% of HIV+ women will be eligible and receive ART [16]. Therefore, at any one time when a cross sectional snapshot is taken, the proportion of HIV+ pregnant women expected to be on ARV or ART will be 52%. This study finds that estimated ARV coverage in the country for mothers is 11.6% and for babies 8.4%, which is below a quarter of the expected need, indicating poor service coverage. Major reasons accounting for this low coverage are: stigma and discrimination; meager supply of commodities, especially ARV drugs; the culture of poor documentation and reporting; the inability to follow up with pregnant women after they learn their HIV status; poor referral and linkage systems; the quality of the service; and, of course, the long standing challenge to reach HIV positive pregnant women for HIV testing and subsequent ARV prophylaxis [11]. These explanations are consistent with the findings of Thomas M., et. al. in Abidjan, Cote d'Ivoire [17].

Data collected and reported in the public health system across all levels cannot be assumed to be complete and/or accurate enough to track process performance or outcomes for PMTCT care. Only a systematic data evaluation can determine the magnitude of the data reporting failure and guide site-specific improvements in data management. As proposed at another study in South Africa, [18] solutions should be developed and tested to improve data quality.

## Conclusions and Recommendations

This study has revealed that, from 2006 to 2010, the progress of ANC coverage assumes a linear trend. If the same trend is assumed to continue in the coming five years, the universal target of reaching all pregnant women for ANC can be achieved in 2015.

During the five-year study period, there has been a remarkable improvement in the potential coverage of PMTCT services due to rapid increase in the number of PMTCT sites. The number of mothers receiving PMTCT services has also shown significant progress.

However, there was a corresponding increase in ANC service coverage and thus, a demand for PMTCT services. This study showed that approximately one-third of mothers are receiving ANC services in either health centers which do not offer PITC HIV services for pregnant women, or at health posts by HEWs, or rarely in outreach sites. These pregnant women constitute a significant number of missed opportunities for HIV counseling and testing, implying the need for a substantial programmatic approach to spread efforts and make service available to these pregnant women.

Based on the findings, improvement in access and utilization of both ANC and PMTCT services, especially efforts that increase the retention of HIV positive mothers in the continuum of care and enable tracking of such mothers, are suggested. Finally, it is also recommended to analyze and interpret ANC/PMTCT data, and use the findings to better enable programs to make informed decisions and take knowledgeable action at all levels ensuring program challenges are appropriately tackled.

## Conflict of interests

The authors declare that they have no competing interests.

## Authors' contributions

TN participated in the design of the study, analysis of data and write up of the manuscript. YW participated in initiating the concept, interpretation of the findings and write up of the manuscript. All authors have read and approved the manuscript.
